# Structural elucidation and interaction profiling of a novel bismuth-based organic–inorganic hybrid: (C_8_H_14_N_2_)_3_(BiCl_6_)_2_

**DOI:** 10.1039/d6ra01928e

**Published:** 2026-04-13

**Authors:** Amin Alibi, Nour Elleuch, Jerome Lhoste, Frédiric Amiard, Sergiu Shova, Mohamed Boujelbene

**Affiliations:** a Laboratory of Physico-Chemistry of Solid State, LR11ES51, Sfax Faculty of Sciences, University of Sfax Sfax 3000 Tunisia m_boujelbene2010@yahoo.fr; b MMM-UMR 6283 CNRS, Lunam, Faculty of Sciences and Techniques, University of Maine Avenue Olivier Messiaen Le Mans Cedex 9 72085 France; c “Petru Poni” Institute of Macromolecular Chemistry Alea Grigore Ghica Voda 41-A 700487 Iasi Romania

## Abstract

This study reports a combined experimental and theoretical investigation of the novel zero-dimensional (0D) bismuth-based hybrid (C_8_H_14_N_2_)_3_(BiCl_6_)_2_, crystallizing in the monoclinic *P*2_1_/*n* space group. Single-crystal X-ray diffraction reveals discrete [BiCl_6_]^3−^ octahedra stabilized within an organized cationic framework through N–H⋯Cl hydrogen bonding, Cl⋯Cl contacts, and π–π stacking. Hirshfeld surface analysis confirms the dominant role of these non-covalent interactions in lattice cohesion, while FT-IR spectroscopy, supported by DFT calculations, validates the vibrational features of the organic cations. Molecular Electrostatic Potential (MEP), Reduced Density Gradient (RDG-NCI), and Electron Localization Function (ELF/LOL) analyses further elucidate electrostatic interactions, dispersion forces, and electron localization. Overall, the results provide comprehensive structural, vibrational, and electronic characterization of (C_8_H_14_N_2_)_3_(BiCl_6_)_2_, offering design insights for stable lead-free bismuth halide hybrids with potential optoelectronic and energy applications.

## Abstract 2

This study reports a combined experimental and theoretical investigation of (C_8_H_14_N_2_)_3_(BiCl_6_)_2_.

## Graphical abstract

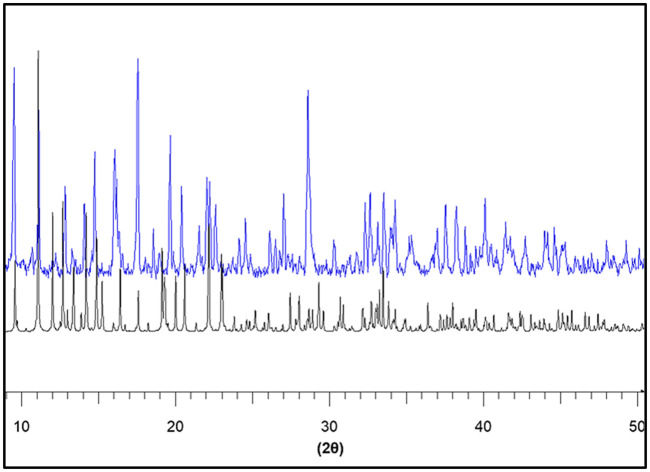

## 1. Introduction

The continuous advancement of hybrid organic–inorganic materials has propelled the discovery of novel functional materials with exceptional optoelectronic, dielectric, and catalytic properties.^1,2^ Among these, halide-based hybrids incorporating ns^2^ metal ions, such as bismuth (Bi^3+^) and antimony (Sb^3+^), have garnered considerable attention due to their remarkable structural tunability and multifunctional properties.^3^ These materials are particularly attractive because they combine the advantages of organic and inorganic components, leading to enhanced structural diversity and tunable physical characteristics.^4^ Bismuth-based halide hybrids, in particular, have emerged as promising candidates for sustainable, lead-free alternatives to traditional perovskites, exhibiting unique electronic structures, strong spin–orbit coupling effects, and notable environmental stability.^5^

Within this family, zero and one-dimensional (0D and 1D) bismuth halide hybrids represent an intriguing subset due to their distinctive chain-like arrangements, which influence charge transport, optical absorption, and intermolecular interactions.^6^ These materials demonstrate significant potential in photovoltaics, nonlinear optics, photodetectors, and other optoelectronic applications.^7,8^ However, despite their promising attributes, several fundamental challenges remain in understanding the intricate relationships between their structure, stability, and functional properties.^9^ The influence of non-covalent interactions, including hydrogen bonding, halogen bonding, and π–π stacking, on the physicochemical properties of these hybrids remains an area of active investigation.^10^

In this work, we introduce (C_8_H_14_N_2_)_3_(BiCl_6_)_2_, a novel bismuth-based hybrid material featuring a zero-dimensional (0D) BiCl_6_-based framework. This material is distinguished by its highly organized supramolecular assembly, in which the discrete BiCl_6_ octahedra are stabilized through extensive non-covalent interactions.^11,12^ The synergy between the organic cationic framework and the inorganic anionic sublattice plays a crucial role in dictating the stability and optical performance of the compound, making it a promising candidate for future applications in advanced optoelectronic devices.^13^

The primary objective of this study is to elucidate the intricate structural and electronic attributes of (C_8_H_14_N_2_)_3_(BiCl_6_)_2_ using a combination of experimental and computational techniques. Single-crystal X-ray diffraction (SC-XRD) is employed to precisely determine the molecular arrangement and intermolecular interactions,^14^ while Hirshfeld surface analysis provides a quantitative assessment of the dominant non-covalent interactions governing the material's stability. Additionally, the vibrational spectroscopy technique, Fourier-transform infrared (FT-IR), is utilized to probe the bonding environment and lattice dynamics of the compound. To complement the experimental findings, density functional theory (DFT) calculations were performed.

Despite the growing interest in hybrid bismuth halides, key knowledge gaps persist in optimizing their electronic and structural properties.^8^ The role of supramolecular interactions in stabilizing the crystal lattice and modulating its optical characteristics remains underexplored.^14^ Furthermore, understanding the impact of ligand coordination environments on the electronic band structure and charge carrier dynamics is crucial for tailoring material performance.^15^ Our study addresses these challenges by providing a comprehensive investigation into the structural and electronic properties of (C_8_H_14_N_2_)_3_(BiCl_6_)_2_, paving the way for rational design strategies aimed at enhancing the functionality of 0D and 1D hybrid materials.^6,11^

Beyond fundamental insights, this research contributes to the broader effort of developing sustainable and lead-free hybrid materials for real-world applications.^5^ The integration of Bi^3+^ as the metal center offers an environmentally benign alternative to lead-based perovskites, aligning with the global push toward green energy solutions.^4,5^ The ability to fine-tune the optoelectronic properties of these hybrids through compositional and structural modifications opens new opportunities for designing high-performance materials for next-generation optoelectronic and sensing applications.^7,13^

This work not only expands the understanding of structure–property relationships in 0D hybrid bismuth halides but also provides a framework for future explorations into their functional versatility.^14,15^ The findings presented herein serve as a stepping stone for the rational design of novel hybrid materials with tailored properties, reinforcing their potential for applications in energy storage, light-emitting devices, and beyond.^16,17^

## 2. Experimental

### 2.1. Materials and characterization techniques

A novel hybrid material was synthesized using BiCl_3_, concentrated HCl (38%), and the commercially available organic molecule C_8_H_12_N_2_, without any additional purification of the reagents. Vibrational spectroscopic studies were conducted to elucidate its structural characteristics. The Fourier transform infrared (FT-IR) absorption spectrum was recorded at room temperature over the spectral range of 3300–500 cm^−1^ using a PerkinElmer FT-IR Paragon 1000 PC spectrometer.

### 2.2. Synthesis of (C_8_H_14_N_2_)_3_(BiCl_6_)_2_

The synthesis of (C_8_H_14_N_2_)_3_(BiCl_6_)_2_ was achieved by dissolving 4-ethylaminomethylpyridine (C_8_H_12_N_2_) and BiCl_3_ (99.95% purity) in 10 mL of distilled water. Following complete dissolution, a few drops of concentrated hydrochloric acid (38% HCl) were introduced into the solution. The mixture was left undisturbed for four days, allowing single crystals to grow gradually through slow evaporation. A well-formed crystal was carefully selected for detailed structural analysis using single-crystal X-ray diffraction (SC-XRD).

**Fig. 1:**
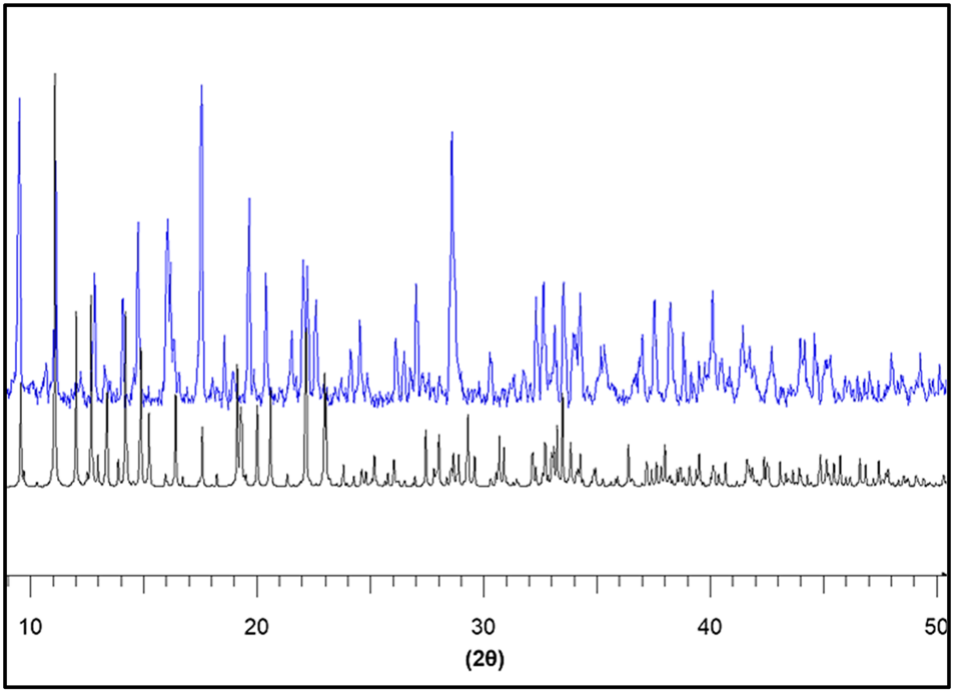
Powder X-ray Diffraction (PXRD) patterns comparing the experimental (blue) and simulated (black) profiles of the (C_8_H_14_N_2_)_3_(BiCl_6_)_2_ compound.

Complementary phase verification of the hybrid compound (C_8_H_14_N_2_)_3_(BiCl_6_)_2_ was carried out by powder X-ray diffraction (Fig. 1), and the resulting diffractogram confirms the structural integrity deduced from single-crystal analysis. The experimental PXRD pattern exhibits sharp, intense, and well-resolved Bragg reflections, demonstrating the high crystallinity and long-range periodic order of the bulk material. A direct comparison with the simulated pattern generated from the refined single-crystal structural model shows an excellent superposition of peak positions, relative intensities, and overall profile features. This strong correspondence unequivocally indicates phase purity, rules out the presence of secondary crystalline impurities, and confirms that the synthesized bulk sample adopts the same structural arrangement as determined crystallographically. The absence of additional unidentified reflections further substantiates the homogeneity of the material and validates the structural model of this zero-dimensional organic–inorganic hybrid composed of discrete [BiCl_6_]^3−^ octahedral units charge-balanced by three doubly protonated C_8_H_14_N_2_^2+^ organic cations, thereby confirming the successful formation of the targeted chlorobismuthate framework.

### 2.3. Single-crystal X-ray data collection and structure determination

The crystallographic structure of (C_8_H_14_N_2_)_3_(BiCl_6_)_2_ was determined through single-crystal X-ray diffraction analysis at a temperature of 293 K. The data collection was performed using an XtaLAB Synergy Dualflex HyPix diffractometer, utilizing Mo-Kα radiation (*λ* = 0.71073 Å). A high-quality crystal with dimensions of 0.17 × 0.13 × 0.08 mm^3^ was selected for the analysis. Structural refinement was carried out using the Olex2-1.5 software package,^18^ with the structure being solved by direct methods and further refined through full-matrix least-squares refinement on F^2^, employing SHELXT 2018/2 (ref. 19) and SHELXL 2018/3.^20^ Anisotropic displacement parameters were applied to all non-hydrogen atoms, while hydrogen atoms were positioned using the HFIX constraint. The CH_2_ and NH_2_ groups were assigned refinement values of 23, CH_3_ was set to 137, while NH and CH groups within the cyclic framework were assigned values of 43. Crystal structure projections and intermolecular packing interactions were visualized using DIAMOND software.^21^ The final refinement yielded *R*_1_ = 0.0218 and w*R*_2_ = 0.0444, with a comprehensive summary of the refinement conditions and structural resolution parameters provided in Table 1.

**Table 1:** Summary of crystal data and structure refinement details

Crystallographic data	
Empirical formula	(C_8_H_14_N_2_)_3_(BiCl_6_)_2_
Color/shape	Clear light colorless, block
Mass molar (g mol^−1^)	1257.99
Temperature (K)	293
Calculated density (Mg m^−3^)	1.846
Crystal system	Monoclinic
Space group	*P*2_1_/*n*
*Z*	4
Unit cell parameters	
*a* (Å)	15.1677 (3)
*b* (Å)	11.9461 (2)
*c* (Å)	25.6539 (5)
*β* (°)	103.092 (2)
Absorption coefficient (mm^−1^)	8.49
Crystal size [mm]^3^	0.17 × 0.13 × 0.08
Number of reflections measured variation of *h*, *k*, *l*	*h* = −18 → 18, *k* = −14 → 14, *l* = −30 → 30
Scanning range of *θ* (°)	2.5 → 25
*F*(000)	2392
Independent parameters	400
*R*_1_	0.0218
w*R*_2_	0.0444
*S* = GooF	1.05
CCDC	2432091

### 2.4. Hirshfeld surface analysis

Hirshfeld surface analysis^22–29^ is a powerful method for identifying and visualizing various non-covalent interactions^30,31^ within a crystalline material, including hydrogen bonding, π–π stacking, and halogen-based contacts. This approach partitions the electron density of a crystal into distinct molecular fragments, providing a detailed examination of intermolecular interactions. The analysis was conducted using the structural data from the crystallographic information file (CIF) and processed through CrystalExplorer 21.5 software.^32^ Furthermore, 2D fingerprint plots were generated to quantify and differentiate the relative contributions of each interaction type.

At any given point on the Hirshfeld surface, two critical distances are defined: *d*_*i*_, representing the shortest distance from the surface to the closest nucleus inside the molecular boundary, and *d*_*e*_, denoting the shortest distance from the surface to the nearest external nucleus. To provide a comparative measure of interaction strength, the normalized contact distance (*d*_norm_) is computed as the sum of the normalized *d*_*i*_ and *d*_*e*_ values, following the equation:1
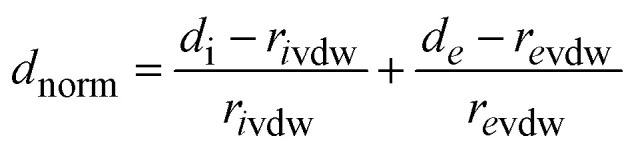
where *r*_*i*vdw_ and *r*_*e*_ᵥ_dw_ correspond to the van der Waals radii of the interacting atoms.^33,34^ This calculated parameter enables the identification of significant intermolecular contacts by highlighting regions of close atomic proximity, providing crucial insights into the packing efficiency and stability of the crystal structure.

### 2.5. Computational details

We carried out a theoretical investigation to achieve precise molecular geometry optimization and rigorously confirm the structural stability of the compound. This study encompassed both geometry optimization and vibrational frequency analysis, ensuring a highly accurate representation of the optimized structure. Computational calculations were performed within the framework of density functional theory (DFT), employing the B3LYP hybrid functional^35^ in conjunction with the LanL2DZ basis set,^36^ as implemented in the Gaussian 09W software package.^37^

To comprehensively account for intermolecular interactions and their effects on both geometric parameters and vibrational properties, we designed a representative cluster model, incorporating three organic cations (C_8_H_14_N_2_)^2+^ and two [BiCl_6_]^3−^ anions. All structural parameters were fully relaxed, ensuring that the system reached a well-defined optimized geometry, with strict convergence criteria applied throughout the process. The vibrational modes were assigned primarily based on DFT-calculated frequencies, with additional verification through detailed visual inspection of animated vibrational modes using GaussView 6.0.16 software.^38^ This approach provided a deep insight into the molecular dynamics and confirmed the structural integrity of the optimized configuration.

## 3. Results and discussion

### 3.1. Crystal structure

**Fig. 2:**
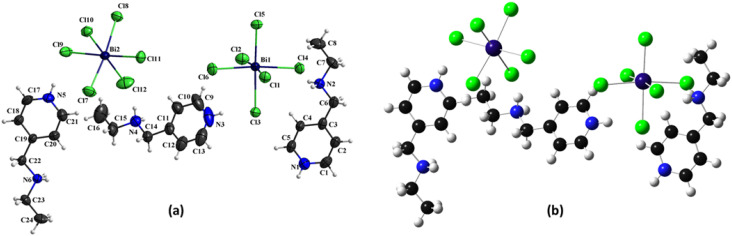
(a) Experimental, and (b) theoretical formula units.

The crystal structure of (C_8_H_14_N_2_)_3_(BiCl_6_)_2_ crystallizes in the monoclinic *P*2_1_/*n* space group with lattice parameters *a* = 15.1677 (3) Å, *b* = 11.9461 (2) Å, *c* = 25.6539 (5) Å, and *β* = 103.092 (2)°, giving a unit cell volume of 4527.53 (15) Å^3^. The asymmetric unit comprises three independent organic cations and two distinct [BiCl_6_]^3−^ octahedra, forming a zero-dimensional (0D) molecular crystal in which discrete ionic species are linked exclusively through non-covalent interactions rather than extended coordination networks (Fig. 2). The calculated density of 1.846 Mg m^−3^ indicates relatively compact packing, governed primarily by electrostatic attractions, hydrogen bonding, and weak supramolecular contacts.

In the absence of packing forces, the optimized model shows minor reorientation of the organic cations and [BiCl_6_]^3−^ units. Nevertheless, the intrinsic geometry of the bismuth-centered octahedra remains essentially unchanged, with Bi–Cl bond lengths and coordination environments well preserved in both representations. The experimental structure benefits from additional stabilization through hydrogen bonding, halogen–halogen contacts, and π–π stacking interactions that are not captured in the isolated theoretical model. These long-range intermolecular forces subtly adjust the spatial distribution of ions and enhance overall lattice stability. Despite minor positional deviations, the core molecular framework, particularly the integrity of the [BiCl_6_]^3−^ octahedra, remains consistent, confirming that the theoretical approach accurately reproduces the fundamental structural features while the experimental model incorporates the collective effects of crystal packing in the final 0D assembly.

**Fig. 3:**
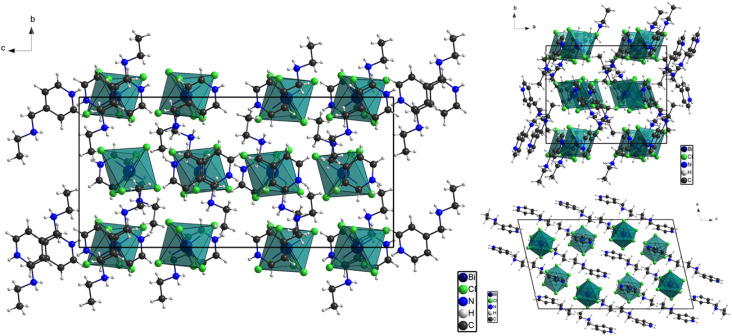
Projections of the unit cell along the three axes.

The unit cell projections along the *a*-, *b*-, and *c*-axes in Fig. 3, provide a comprehensive visualization of the molecular arrangement in the 0D hybrid organic–inorganic crystal (C_8_H_14_N_2_)_3_(BiCl_6_)_2_. As a zero-dimensional system, the structure consists of discrete [BiCl_6_]^3−^ octahedral anions and organic cations that do not form extended coordination or covalent networks but instead rely on electrostatic forces, hydrogen bonding, halogen interactions, and supramolecular packing forces for structural stability. The inversion center, as the primary symmetry element, dictates the spatial organization of the molecular components, ensuring a periodic and symmetrical distribution of the anionic and cationic entities within the unit cell.

The *a*-axis projection reveals the separation between the discrete [BiCl_6_]^3−^ anions, which are symmetrically distributed throughout the structure. Each Bi-centered octahedron is surrounded by organic cations, which maintain the electrostatic charge balance and prevent direct Bi⋯Bi interactions, reinforcing the isolated nature of the anionic entities. The cations adopt a staggered arrangement, optimizing the spatial packing and maximizing their hydrogen bonding interactions with the halide ligands of the [BiCl_6_]^3−^ units. The projection also confirms that no extended covalent linkages exist between neighboring octahedra, preserving the discrete molecular nature of the compound.

The *b*-axis projection highlights the stacking of organic cations between the [BiCl_6_]^3−^ anions, which plays a crucial role in the packing efficiency and stability of the crystal. The organic cations are arranged in a way that facilitates π–π stacking interactions (discussed in Fig. 2S) as well as C–H⋯π interactions, ensuring a compact assembly. Additionally, the positioning of the [BiCl_6_]^3−^ octahedra in relation to the organic cations allows for optimal halogen bonding interactions, which contribute to the stabilization of the crystalline lattice.

From the *c*-axis perspective, the periodicity of the structure is evident, demonstrating a well-ordered, repeating arrangement of the cations and anions. The organic cations effectively separate the [BiCl_6_]^3−^ octahedra, preventing any direct halide bridging or metal–metal interactions, which is a defining characteristic of 0D hybrid material-related structures. The inversion symmetry ensures that each unit cell maintains a balanced charge distribution and optimized spatial configuration, contributing to the overall structural stability.

The structural framework of (C_8_H_14_N_2_)_3_(BiCl_6_)_2_ exhibits a well-defined halogen–halogen interaction network in which the spatial distribution of chlorine atoms contributes significantly to lattice stabilization. The halide ligands of the two independent [BiCl_6_]^3−^ octahedra participate in several Cl⋯Cl contacts, forming an organized halogen-bonding scheme that reinforces cohesion within the zero-dimensional (0D) molecular crystal. These non-covalent interactions help maintain the ordered arrangement of the discrete octahedral anions.

**Fig. 4:**
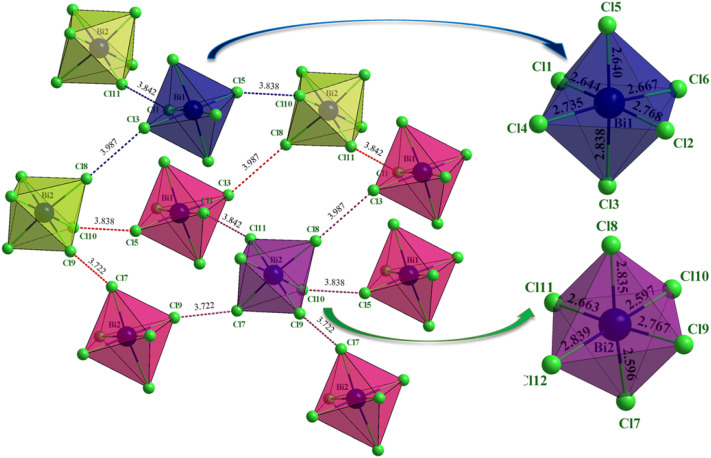
Environments of the two anionic entities and their distortions.

As illustrated in Fig. 4, the Bi2 octahedron (violet) participates in five distinct Cl⋯Cl interactions with neighboring units, whereas each Bi1 octahedron (blue) is involved in only three such contacts. This difference indicates an asymmetric contribution of the two independent [BiCl_6_]^3−^ units to supramolecular stabilization, likely governed by packing constraints and octahedral orientation. The interacting Cl⋯Cl distances fall within the typical range of non-covalent halogen bonds and correspond to Type-II interactions characterized by directional σ-hole attraction (≈160–180°). The greater number of contacts around Bi2 therefore suggests stronger local stabilization compared with the Bi1 environment.

Collectively, these halogen–halogen interactions regulate the relative positioning of the octahedra, ensuring separation without direct Bi⋯Bi contacts and preserving the integrity of the [BiCl_6_]^3−^ coordination geometry (Fig. 1S). The Bi2 unit, involved in five contacts, lies within a more densely stabilized region of the lattice, while the Bi1 unit retains slightly greater spatial freedom. This controlled asymmetry prevents excessive rigidity and favors efficient packing within the crystal.

The [BiCl_6_]^3−^ anionic units maintain a well-defined octahedral coordination geometry in which each Bi^3+^ center is surrounded by six chloride ligands. Despite the differences in their halogen interaction environments, both Bi1 and Bi2 octahedra remain structurally stable, showing no significant distortions or elongations in their Bi–Cl coordination bonds, thus preserving the integrity of the isolated inorganic units.

To quantify the deviation of the [BiCl_6_]^3−^ octahedra from an ideal octahedral geometry, a distortion index (ID) was calculated based on the variations in Bi–Cl bond lengths across each octahedron:2
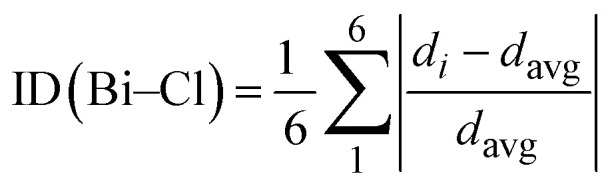
where *d*_*i*_ (*i* = 1 to 6) represent the individual Bi–Cl bond lengths, and *d*_avg_ is the mean bond length within each octahedral unit. This calculation provides a quantitative measure of structural deviation, allowing for a direct assessment of any distortions induced by packing constraints, halogen interactions, or electronic effects.3
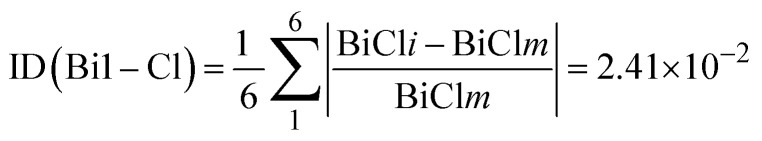
4
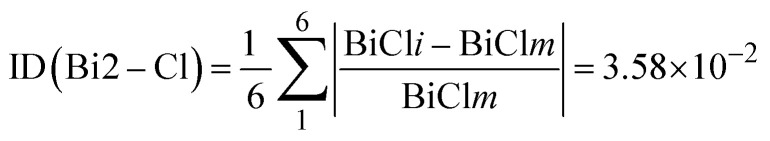


Bi1 octahedron distortion index: 0.0241 (2.41%), while Bi2 octahedron distortion index: 0.0358 (3.58%). Bi2 exhibits a slightly higher distortion than Bi1, suggesting greater deviation from an ideal octahedral geometry, which may be due to its involvement in five halogen–halogen interactions *versus* three for Bi1.

Analysis of bond lengths indicates that both Bi1 and Bi2 octahedra exhibit negligible distortion, confirming a highly regular coordination environment. No significant bond elongation or angular deviation is observed, demonstrating that steric effects and lattice strain do not perturb the anionic units. The stereochemically inactive 6s^2^ lone pair of Bi^3+^ does not induce measurable asymmetry, further supporting the preservation of nearly ideal octahedral geometry. Retention of this geometry is crucial for the packing efficiency and stability of the 0D crystal. Halogen bonding (Fig. 4 and 1S), π–π stacking (Fig. 2S), and hydrogen bonding (Fig. 3S) collectively ensure that the discrete octahedra remain electronically and structurally isolated yet firmly stabilized, with no excessive packing stress or lattice distortion.

Such geometric regularity also impacts optical properties. In hybrid metal–halide systems, octahedral distortions typically influence band structure and charge transport. Here, minimal deviation from ideal coordination suggests that electronic transitions in the Bi–Cl framework are dominated by intrinsic electronic factors, enabling predictable optoelectronic behavior.

The organic cations adopt a well-organized supramolecular stacking arrangement. Four central cations engage in offset parallel-displaced π–π stacking between aromatic rings, maximizing π-cloud overlap while limiting steric repulsion. Interplanar separations indicate moderate to strong interactions, reinforcing rigidity and cohesion in the organic sublattice, reducing void space, and potentially facilitating limited electronic delocalization. The two terminal cations primarily participate in weaker C–H⋯π contacts between aliphatic hydrogens and neighboring aromatic π systems, refining molecular orientation and contributing additional stabilization. Together with halogen and hydrogen bonding, these interactions form a complementary non-covalent network governing supramolecular assembly and structural integrity.

Halogen bonding is directional and electrostatically driven, stabilizing the anionic [BiCl_6_]^3−^ framework; π–π stacking arises from aromatic overlap and consolidates cation–cation cohesion; hydrogen bonding provides localized bridges between cations and anions. The central π-stacked cations establish a robust electronically stabilized network, while peripheral C–H⋯π contacts provide further anchoring. This hierarchical arrangement enhances mechanical rigidity, limits molecular mobility, and may subtly influence dielectric behavior.

Hydrogen bonding plays a decisive role in linking discrete [BiCl_6_]^3−^ octahedra with surrounding cations. Multiple N–H⋯Cl interactions form an ordered network compensating for the lack of covalent connectivity, reinforcing electrostatic stabilization. Donor–acceptor distances range from 3.049 to 3.392 Å, with H⋯Cl separations of 2.19–2.53 Å, consistent with moderate to strong bonds. The shortest, N5–H5⋯Cl9 (2.19 Å; 178°), is highly linear and represents the strongest interaction, while others such as N3–H3⋯Cl3 (3.062 Å; 166°) and N4–H4B⋯Cl12 (3.167 Å; 158°) further reinforce the network. Bond angles spanning 149–178° highlight the directional nature of these interactions.

Empirical correlations suggest that nearly linear hydrogen bonds (<2.2 Å; >165°) contribute 15–30 kJ mol^−1^, moderate contacts (2.3–2.5 Å; 150–165°) 10–15 kJ mol^−1^, and weaker interactions <10 kJ mol^−1^. Accordingly, the strongest N–H⋯Cl bonds act as primary short-range stabilizing forces, while longer contacts provide secondary reinforcement. Compared with Cl⋯Cl halogen interactions, hydrogen bonds are stronger and structurally defining, directly bridging organic and inorganic components. Halogen and π–π interactions complement this network, producing a cooperative stabilization scheme that minimizes lattice distortion, prevents molecular disorder, and enhances the structural and thermal stability of the crystal (Table 2).

**Table 2:** Interatomic distances and hydrogen bond angles of the compound^a^

D–H···A	D–H (Å)	H⋯A (Å)	D⋯A (Å)	D–H···A (°)
N5–H5⋯Cl9	0.86	2.19	3.049 (5)	178
N6–H6A⋯Cl8i	0.89	2.39	3.187 (5)	149
N6–H6B⋯Cl5ii	0.89	2.53	3.392 (5)	165
N2–H2A⋯Cl11iii	0.89	2.48	3.357 (5)	168
N2–H2B⋯Cl4	0.89	2.39	3.191 (5)	150
N3–H3⋯Cl3	0.86	2.22	3.062 (6)	166
N4–H4A⋯Cl3iv	0.89	2.46	3.311 (5)	162
N4–H4B⋯Cl12	0.89	2.32	3.167 (5)	158

a Symmetry codes: (i) −*x* + 3/2, *y* − 1/2, −*z* + 3/2; (ii) *x* − 1/2, −*y* + 3/2, *z* + 1/2; (iii) −*x* + 2, −*y* + 1, −*z* + 1; (iv) −*x* + 1, −*y* + 1, −*z* + 1.

### 3.2. Vibrational study

**Fig. 5:**
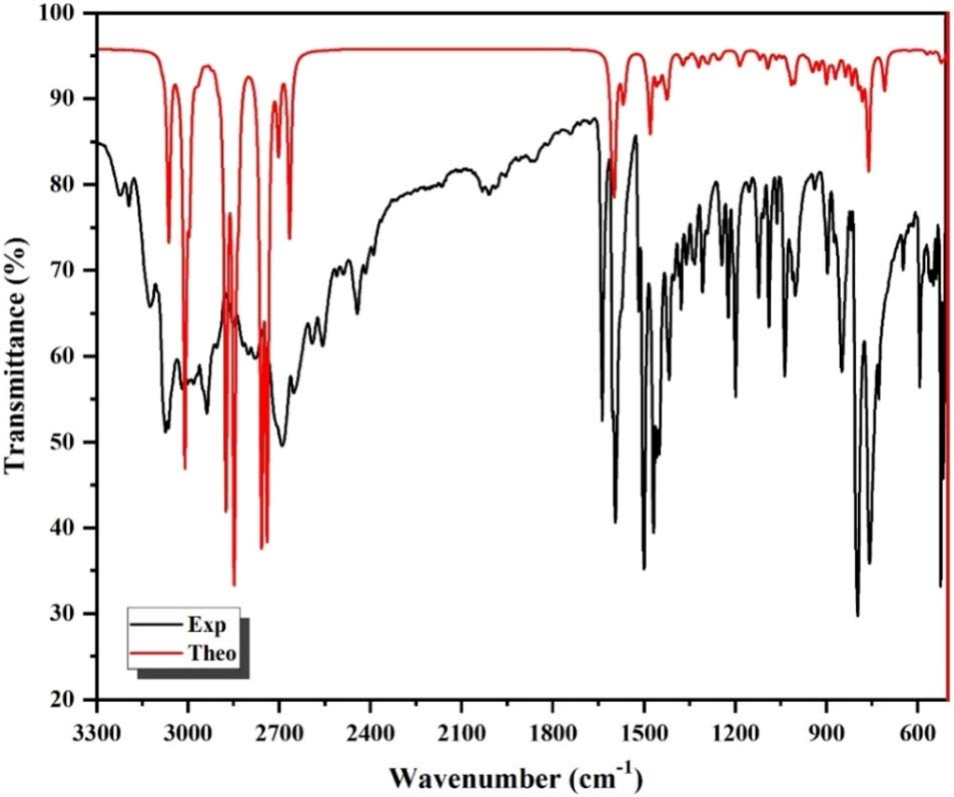
FTIR spectrum of the titled compound.

**Fig. 6:**
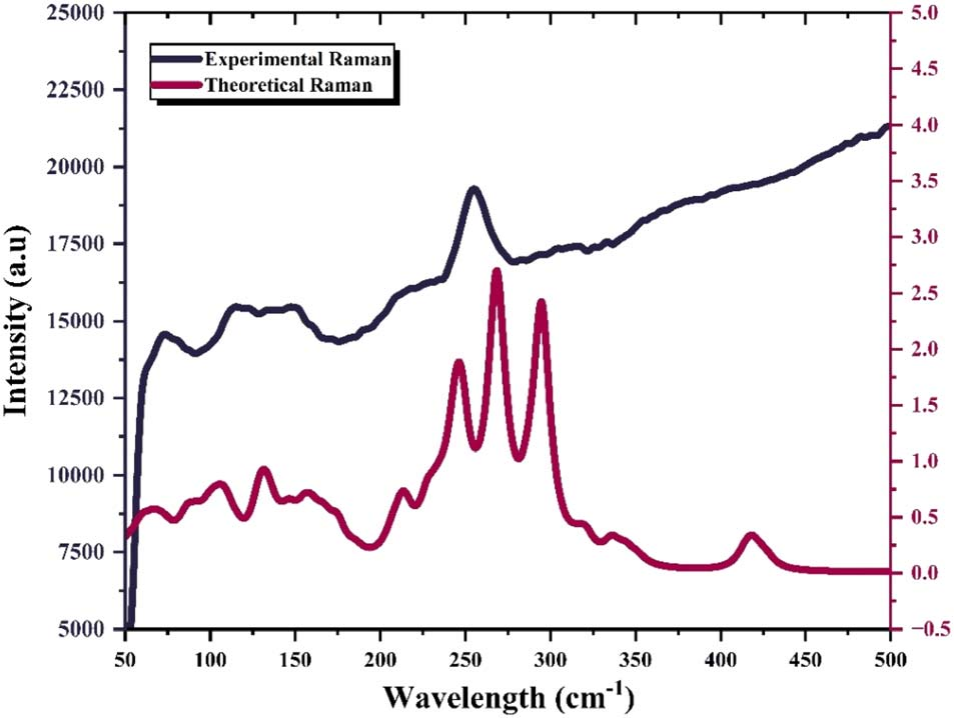
Raman spectrum of the titled compound.

Fourier Transform Infrared (FTIR) and Raman spectroscopy were performed at room temperature to characterize the vibrational properties of (C_8_H_14_N_2_)_3_(BiCl_6_)_2_. FTIR mainly probes the organic (C_8_H_14_N_2_)^2+^ cations, as the heavy [BiCl_6_]^3−^ units exhibit weak infrared activity due to the large atomic masses of Bi and Cl. In contrast, Raman spectroscopy efficiently detects the metal–halide stretching and bending modes of the inorganic octahedra. To ensure reliable vibrational assignments, Density Functional Theory (DFT) calculations were performed, enabling direct comparison between experimental and theoretical frequencies. The experimental and calculated FTIR spectra are presented in Fig. 5, while the Raman spectra appear in Fig. 6. The detailed vibrational assignments are summarized in Table 3. The strong agreement between calculated and experimental frequencies validates the spectral interpretation and supports the crystallographic structural model. Although periodic DFT could account for lattice effects, the molecular approach provides a physically meaningful description of the vibrational behavior of this zero-dimensional chlorobismuthate system.

**Table 3:** The attributions of calculated and observed frequencies of the vibration modes of the compound^a^

IR (cm^−1^)	Raman (cm^−1^)	Calc. wavenumbers (cm^−1^)	Assignment
3070	—	3066	*ν*_as_(NH_2_)
2985	—	3010	*ν*_as_(CH_2_) +*ν*_as_(CH_3_)
2926	—	2890	*ν*_s_(CH_2_)
2854	—	2877	*ν*_s_(CH_3_)
2810	—	2840	*ν*(N–H)
2647–2780	—	2669–2758	*ν*_s_(NH_2_)
1636	—	1603	*δ*(NH_2_)
1592	—	1566	*ν*(C=C) + *ν*(N=C)
1495	—	1485	*δ*(CH_2_) + *ω*(NH_2_)
1462	—	1455	*τ*(CH_2_) + *τ*(NH_2_)
1414	—	1425	*ω*(CH_2_)
1376	—	1365	*ν*(N–C)
1335	—	1317	*β*_as_(C–H)
1302	—	1291	*β*_s_(C–H)
1243	—	1254	*τ*(CH_3_)
1195	—	1187	*δ*(C–C–N)
1123	—	1121	*ν*_as_(C–C–N)
1086	—	1088	*γ*(N–H)
1016	—	1035	*γ*_as_(C–H)
1001	—	998	*γ*_s_(C–H)
897	—	897	*δ*(C–C–N)
849	—	864	*γ*_s_(C–H)
803	—	787	*γ*(N–H)
753	—	760	*γ*_as_(C–H)
672	—	700	*ω*(C–N–C)
585	—	566	*ω*(C–C–C)
—	254	246–269–294	*ν*_as_(Bi–Cl)
—	211	213	*ν*_s_(Bi–Cl)
—	146	132	*δ*_as_(Cl–Bi–Cl)
—	113	107	*δ*_s_(Cl–Bi–Cl)
—	71	65	Lattice mode

a *ν*_s_: symetric stretching, *ν*_as_: assymetric stretching, *β*: in plane bending, *γ*: out plane bending, *δ*: scissoring, *ρ*: rocking, *ω*: wagging.

The FTIR spectrum recorded between 3300 and 500 cm^−1^ displays the characteristic vibrational modes of the organic framework. In the high-frequency region, N–H stretching vibrations of the protonated amine groups dominate. The asymmetric N–H stretching band appears at 3070 cm^−1^ (calculated 3066 cm^−1^), while symmetric stretching modes occur between 2647 and 2780 cm^−1^, in good agreement with the calculated range of 2669–2758 cm^−1^. The N–H bending vibration at 1636 cm^−1^ (calculated 1603 cm^−1^) confirms protonation and involvement in hydrogen bonding.

C–H stretching vibrations from alkyl and aromatic groups are observed between 2985 and 2854 cm^−1^. The asymmetric CH_3_ stretching band at 2985 cm^−1^ (calculated 3010 cm^−1^), the symmetric CH_3_ mode at 2854 cm^−1^ (calculated 2877 cm^−1^), and CH_2_ stretches at 2926 and 2890 cm^−1^ agree closely with theoretical predictions, confirming the structural integrity of the organic moieties.

In the fingerprint region (1500–500 cm^−1^), C–N stretching vibrations at 1376, 1335, and 1302 cm^−1^ correspond well with calculated values (1365, 1317, and 1291 cm^−1^), indicating preservation of the nitrogen coordination environment. Skeletal C–C–N vibrations at 1195 and 1123 cm^−1^ (calculated 1187 and 1121 cm^−1^) further confirm the rigidity of the molecular framework. Additional features include C–H bending modes at 1016 cm^−1^ (in-plane) and 849 cm^−1^ (out-of-plane), closely matching computed values of 1035 and 864 cm^−1^. Skeletal deformation modes such as *ω*(C–N–C) at 672 cm^−1^ and *ω*(C–C–C) at 585 cm^−1^ also align well with calculated values of 700 and 566 cm^−1^, respectively. Overall, the consistent agreement between experimental and theoretical frequencies confirms the reliability of the vibrational assignments and the stability of the hybrid structure.

The vibrational attributions were further validated through comparison with a previously reported study involving the same organic molecular unit.^39^ A frequency shift of approximately 150 cm^−1^ was observed for modes associated with hydrogen-bond-sensitive functional groups. This shift reflects the increased number and strength of hydrogen bonds in the present compound, which modify intermolecular interactions and enhance structural stability. The strengthened hydrogen-bond network therefore significantly influences the vibrational landscape and molecular organization of this hybrid framework.

The Raman spectrum of (C_8_H_14_N_2_)_3_(BiCl_6_)_2_ is dominated by vibrational features arising from the inorganic [BiCl_6_]^3−^ octahedral units and provides complementary confirmation of the assignments established by FTIR analysis. In the low-wavenumber region characteristic of metal–halide frameworks (50–300 cm^−1^), the most intense feature appears at 254 cm^−1^ and is attributed to the asymmetric stretching mode *ν*_as_(Bi–Cl). Density functional theory calculations predict three closely spaced *ν*_as_(Bi–Cl) modes in the 246–294 cm^−1^ range, originating from the two crystallographically independent [BiCl_6_]^3−^ octahedra present in the asymmetric unit. Experimentally, these calculated components coalesce into a single broadened band, reflecting their near-degenerate nature and the limited spectral resolution between overlapping vibrations, which explains the slight discrepancy between theoretical multiplicity and the observed single-peak profile. The symmetric stretching vibration *ν*_s_(Bi–Cl) is observed at 211 cm^−1^, in excellent agreement with the calculated value of 213 cm^−1^, confirming the preservation of the octahedral coordination environment around Bi^3+^. Bending modes are detected at 146 and 113 cm^−1^, corresponding to the asymmetric *δ*_as_(Cl–Bi–Cl) and symmetric *δ*_s_(Cl–Bi–Cl) deformations, respectively, closely matching the theoretical predictions at 132 and 107 cm^−1^. Finally, a lattice mode appears at 71 cm^−1^, consistent with the calculated value of 65 cm^−1^, and is associated with collective translational and rotational motions of the inorganic octahedra within the crystal lattice. The overall excellent agreement between experimental and computed frequencies, together with the coherent assignment of stretching, bending, and lattice vibrations, unequivocally validates the structural model of discrete [BiCl_6_]^3−^ units and confirms the dynamical stability of the chlorobismuthate framework in this zero-dimensional organic–inorganic hybrid material.

### 3.3. Hirshfeld surface analysis (HSA)

**Fig. 7:**
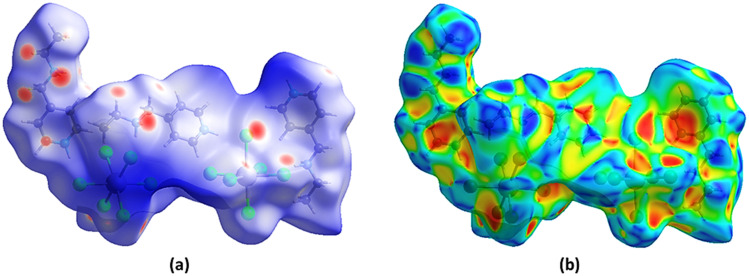
Hirshfeld surface of the compound (C_8_H_14_N_2_)_3_(BiCl_6_)_2_, (a) 3D *d*_norm_ surface, (b) surface index.

Fig. 7 and 4S provide a combined visualization and quantitative analysis of the non-covalent interactions governing the molecular organization of (C_8_H_14_N_2_)_3_(BiCl_6_)_2_. The Hirshfeld surface mapping (Fig. 7) offers a spatial representation of intermolecular contact regions within the crystal, while the 2D fingerprint plots (Fig. 4S) quantify the relative contributions of the different interaction types. Together, these analyses clarify the stabilization mechanisms operating in the lattice and highlight the central role of hydrogen bonding, halogen interactions, and van der Waals forces in determining the packing efficiency and structural stability of the hybrid material.

The Hirshfeld surface mapped with dnorm in Fig. 7 provides a three-dimensional representation of intermolecular contacts in (C_8_H_14_N_2_)_3_(BiCl_6_)_2_. In this mapping, the interaction intensity is color coded according to the normalized contact distance. Red regions correspond to contacts shorter than the sum of van der Waals radii, indicating strong intermolecular interactions such as hydrogen bonding or halogen contacts. White regions represent contacts close to van der Waals distances and therefore correspond to moderate intermolecular interactions. Blue regions indicate longer intermolecular separations or areas where no significant contacts occur, marking the outer molecular boundaries.

The Hirshfeld surface of (C_8_H_14_N_2_)_3_(BiCl_6_)_2_ is characterized by prominent red areas associated with hydrogen bonding interactions between the N–H groups of the organic cations and the chloride atoms of the [BiCl_6_]^3−^ octahedra. These N–H⋯Cl interactions represent the principal stabilizing force within the crystal, forming a directional network that links the organic and inorganic components. The localization of the red regions around hydrogen bond donors and acceptors highlights the strong electrostatic nature and directional character of these contacts, which contribute significantly to lattice cohesion.

Additional red regions on the Hirshfeld surface correspond to Cl⋯Cl contacts between neighboring inorganic units. Although weaker than hydrogen bonds, these halogen interactions contribute to optimizing the packing arrangement and reinforcing supramolecular stability. The Type-II halogen bonding present in this structure arises from σ-hole electrostatic interactions between polarized chlorine atoms and promotes cohesion between adjacent [BiCl_6_]^3−^ units.

White and faint blue regions on the Hirshfeld surface indicate weaker intermolecular contacts, mainly associated with H⋯H interactions between neighboring organic cations. These van der Waals interactions, while non-directional, contribute to the overall packing efficiency by filling intermolecular space and preventing excessive void formation within the crystal lattice. The combined effect of strong hydrogen bonds, secondary halogen interactions, and weaker dispersion forces leads to a compact and well-organized supramolecular arrangement.

The 2D fingerprint plots presented in Fig. 4S provide a quantitative decomposition of the Hirshfeld surface into individual interaction contributions. The most prominent feature corresponds to H⋯Cl contacts, which appear as sharp spikes at low *d*_*i*_ and *d*_*e*_ distances. These spikes reflect the strong and directional N–H⋯Cl hydrogen bonds linking the organic cations to the [BiCl_6_]^3−^ octahedra, confirming their dominant role in stabilizing the structure.

A secondary region observed at moderate *d*_*i*_ and *d*_*e*_ distances corresponds to Cl⋯Cl interactions, confirming the presence of halogen contacts that complement hydrogen bonding and help maintain the structural alignment of the inorganic units. In contrast, the broader distribution in the fingerprint plots corresponds to H⋯H contacts, characteristic of van der Waals interactions between organic moieties. These dispersion-driven contacts contribute to the fine adjustment of molecular packing and help maintain structural order within the lattice.

By analyzing the relative area contributions of each interaction type, it becomes evident that hydrogen bonding accounts for the majority of intermolecular stabilization, while halogen interactions and van der Waals forces provide additional support in maintaining the structural integrity of the crystal. The sharp, well-defined peaks in the fingerprint plots reinforce the highly ordered nature of the lattice, confirming that (C_8_H_14_N_2_)_3_(BiCl_6_)_2_ adopts an optimized molecular arrangement where non-covalent forces work in concert to establish a stable and densely packed hybrid material.

The Hirshfeld surface mapping and 2D fingerprint plot analysis unequivocally demonstrate that the structural organization of (C_8_H_14_N_2_)_3_(BiCl_6_)_2_ is governed by a delicate balance of strong hydrogen bonding, stabilizing halogen interactions, and weak van der Waals forces. The hydrogen bonding network, particularly N–H⋯Cl interactions, plays the most dominant role in maintaining lattice cohesion and electrostatic charge balance. Meanwhile, halogen bonding subtly influences the packing arrangement and molecular orientation, ensuring a compact and thermodynamically stable structure. The secondary contribution of van der Waals forces further refines the packing efficiency, preventing steric clashes and maintaining the mechanical integrity of the crystal lattice.

### 3.4. Non-covalent interactions analysis

A detailed description of the electronic structure and interaction landscape of the hybrid compound (C_8_H_14_N_2_)_3_(BiCl_6_)_2_ was obtained using complementary Density Functional Theory (DFT) analyses, including Molecular Electrostatic Potential (MEP), Non-Covalent Interaction Reduced Density Gradient (NCI-RDG), Electron Localization Function (ELF), and Localized Orbital Locator (LOL) mappings. Together, these methods characterize the charge distribution, orbital features, and non-covalent interactions governing the compound's structural stability and reactivity.

#### 3.4.1. Molecular electrostatic potential MEP analysis

**Fig. 8:**
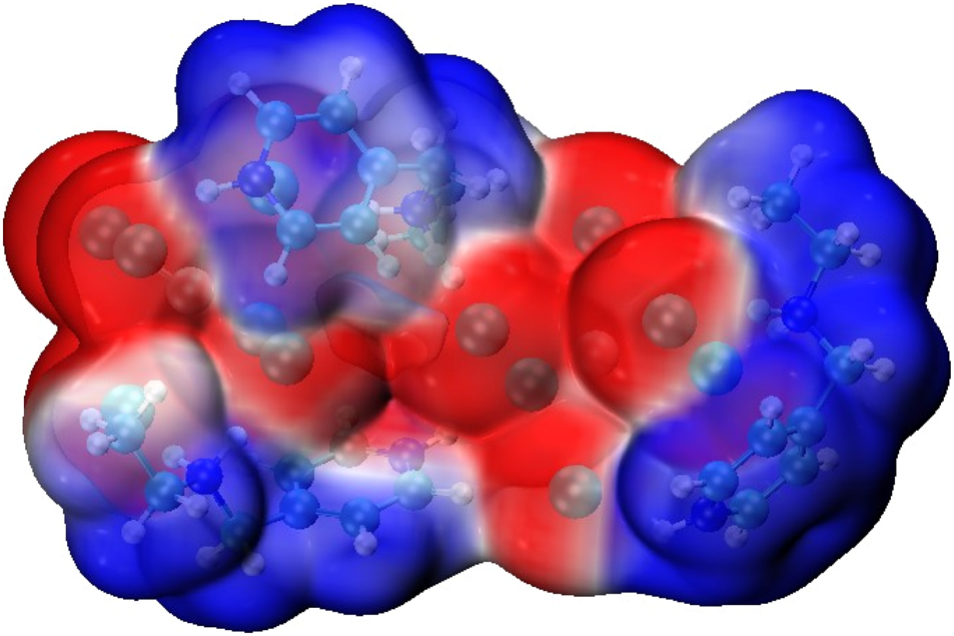
Molecular electrostatic potential analysis of the target compound.

The Molecular Electrostatic Potential (MEP) analysis of (C_8_H_14_N_2_)_3_(BiCl_6_)_2_, presented in Fig. 8, provides a detailed visualization of the compound's surface charge distribution, revealing key aspects of its reactive behavior and intermolecular interaction potential. The MEP map clearly distinguishes regions of negative and positive electrostatic potential, corresponding to nucleophilic and electrophilic sites, respectively. These regions play a major role in governing the non-covalent interactions responsible for lattice cohesion and supramolecular stability.

In this structure, the most negative potential regions (red) are localized around the chloride ligands of the [BiCl_6_]^3−^ anions, indicating strong nucleophilic character. These sites are likely involved in electrostatic and hydrogen-bonding interactions with the surrounding protonated organic cations. In contrast, the positive potential regions (blue), located around the N–H bonds of the (C_8_H_14_N_2_)^2+^ cations, confirm their function as hydrogen bond donors. The MEP surface therefore illustrates a well-defined electrostatic complementarity guiding the arrangement and interaction of the cationic and anionic components.

The π-systems of the organic moieties exhibit relatively neutral potential, suggesting partial electron delocalization and a possible contribution to weak π–π interactions within the solid-state framework. Overall, the MEP analysis highlights the key intermolecular interaction sites and confirms the role of electrostatic complementarity in stabilizing the structure of (C_8_H_14_N_2_)_3_(BiCl_6_)_2_.

#### 3.4.2. Non-covalent interaction NCI-RDG analysis

**Fig. 9:**
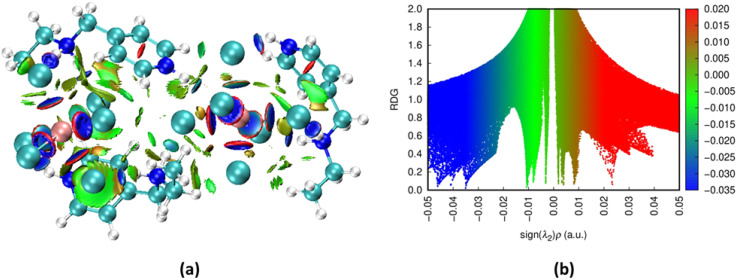
NCI (a) and RDG (b) visualization of the target compound.

The Non-Covalent Interaction (NCI) analysis based on the Reduced Density Gradient (RDG) framework provides a visualization of the weak interactions stabilizing the crystal structure of (C_8_H_14_N_2_)_3_(BiCl_6_)_2_ (Fig. 9). These interactions, including hydrogen bonding, van der Waals forces, and steric effects, govern the supramolecular organization of the compound, mainly arising from interactions between the protonated organic cations and the inorganic [BiCl_6_]^3−^ anions.

The NCI isosurface representation displays green regions around the chloride atoms of the [BiCl_6_]^3−^ anions and the neighboring N–H groups of the organic cations, indicating moderate stabilizing hydrogen bonds that maintain electrostatic and directional cohesion within the lattice. Additional faint green areas between the alkyl chains of the cations correspond to dispersion (van der Waals) interactions contributing to the packing arrangement of the structure.

The RDG scatter plot supports these observations. Blue regions at negative values of sign(*λ*_2_)*ρ* indicate attractive interactions dominated by hydrogen bonding, while the green region near zero reflects weaker van der Waals contributions. The limited red zones at positive values indicate minimal steric repulsion, confirming the spatial compatibility between the organic and inorganic units.

#### 3.4.3. Electron localization function (ELF) and localized orbital locator (LOL) analyses

**Fig. 10:**
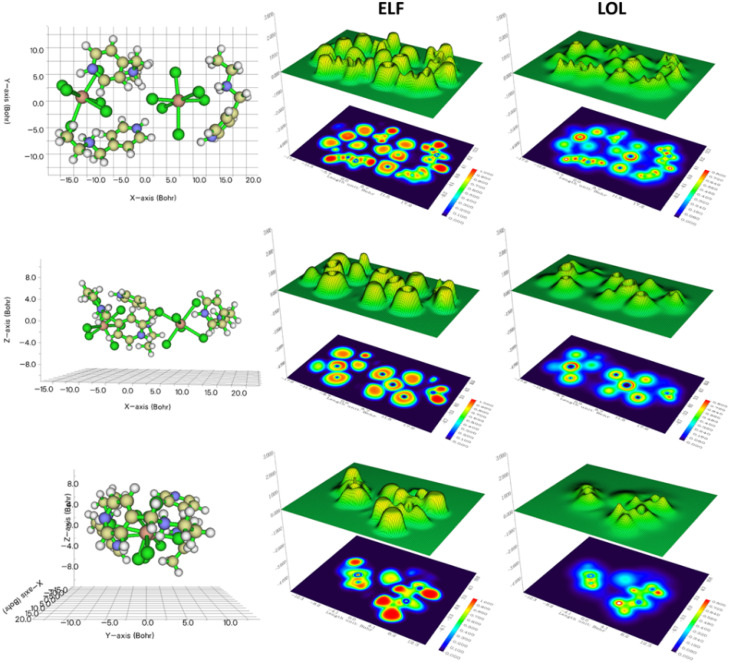
Electron Localization Function (ELF) and Localized Orbital Locator (LOL) visualization in (*XY*), (*XZ*), and (*YZ*) planes.

The Electron Localization Function (ELF) analysis of (C_8_H_14_N_2_)_3_(BiCl_6_)_2_, illustrated in Fig. 10 across the (*XY*), (*XZ*), and (*YZ*) planes (top to bottom), provides detailed insight into the spatial distribution of localized electron density associated with covalent bonds, lone pairs, and potential interaction sites. The ELF maps clearly reveal strong electron localization around the chloride atoms of the [BiCl_6_]^3−^ anions, corresponding to their nonbonding electron pairs and confirming their role as efficient hydrogen-bond acceptors within the crystal lattice.

In the (*XY*) and (*XZ*) projections, pronounced ELF localization is also observed around the nitrogen atoms of the (C_8_H_14_N_2_)^2+^ cations, reflecting their involvement in hydrogen bonding interactions and highlighting their contribution to the stabilization of the supramolecular framework. Moderately localized regions between the organic and inorganic units further indicate the presence of directional electrostatic interactions that reinforce lattice cohesion. When interpreted across the three planes, the ELF distribution illustrates a clear spatial complementarity between the organic cations and the anionic clusters, supporting the ordered arrangement of the structure and correlating with the interaction regions identified in the MEP and NCI analyses.

The Localized Orbital Locator (LOL) analysis of (C_8_H_14_N_2_)_3_(BiCl_6_)_2_, also presented in Fig. 10 along the same planes, complements the ELF results by providing a refined description of orbital localization and the spatial behavior of bonding and nonbonding electrons. Strong LOL localization appears around the chlorine atoms of the [BiCl_6_]^3−^ units, confirming their electron-rich character and their capacity to participate in directional hydrogen bonding with the surrounding organic cations.

High LOL values are likewise observed near the nitrogen atoms of the (C_8_H_14_N_2_)^2+^ cations, particularly in the (*XZ*) and (*YZ*) planes, reflecting localized orbital density associated with lone pairs. Smooth gradient transitions between the organic and inorganic components suggest limited orbital overlap and slight charge delocalization at the interface. Overall, the LOL results reinforce the ELF observations, revealing a balanced distribution of localized orbitals that governs the stability and interaction directionality of the hybrid framework.

## 4. Conclusion

The present study delivers a comprehensive structural, vibrational, and electronic characterization of the novel zero-dimensional bismuth-based hybrid (C_8_H_14_N_2_)_3_(BiCl_6_)_2_. Single-crystal X-ray diffraction (SC-XRD) revealed discrete [BiCl_6_]^3−^ octahedra embedded in an organized organic framework, with stabilization provided by an intricate network of N–H⋯Cl hydrogen bonding, Cl⋯Cl halogen contacts, and π–π stacking. Hirshfeld surface analysis quantified the relative contributions of these interactions, highlighting hydrogen bonding as the dominant force, while halogen interactions and dispersion contacts refined the packing efficiency and reinforced lattice cohesion.

Fourier-transform infrared (FT-IR) spectroscopy, strongly supported by DFT-calculated vibrational frequencies, confirmed the structural integrity of the organic cations and validated the robustness of the supramolecular framework. The excellent correlation between experiment and theory further emphasized the role of non-covalent forces in shaping the vibrational landscape.

Complementary computational analyses provided deeper insights into the interaction topology. Molecular Electrostatic Potential (MEP) mapping pinpointed nucleophilic and electrophilic regions guiding intermolecular recognition; Reduced Density Gradient (RDG-NCI) visualization revealed stabilizing hydrogen bonding and dispersion contributions with negligible steric clashes; and Electron Localization Function (ELF) together with Localized Orbital Locator (LOL) descriptors mapped electron pairing, orbital confinement, and charge localization. Together, these approaches offered a multidimensional perspective on the electronic structure and supramolecular organization of the hybrid material.

By integrating crystallographic, spectroscopic, and theoretical methods, this work demonstrates how the synergy of non-covalent interactions and electronic localization underpins the stability of (C_8_H_14_N_2_)_3_(BiCl_6_)_2_. The minimal distortion of the [BiCl_6_]^3−^ octahedra, combined with dense supramolecular packing, ensures the preservation of the 0D lattice and underscores its potential for optoelectronic, photonic, and energy-related applications. More broadly, these findings provide guiding principles for the design of sustainable, lead-free bismuth halide frameworks, reinforcing the importance of supramolecular engineering and advanced DFT descriptors in tailoring the next generation of functional hybrid materials.

## Conflicts of interest

The authors declare that they have no known competing financial interests or personal relationships that could have appeared to influence the work reported in this paper.

## Supplementary Material

RA-016-D6RA01928E-s001

RA-016-D6RA01928E-s002

## Data Availability

The raw/processed data required to reproduce these findings are available and can be provided if requested. CCDC 2432091 contains the supplementary crystallographic data for this paper.^40^ Supplementary information (SI) is available. See DOI: https://doi.org/10.1039/d6ra01928e.
