# Supplementary material for: Structural elucidation and interaction profiling of a novel bismuth-based organic–inorganic hybrid: (C8H14N2)3(BiCl6)2
Source: RSC Adv. 2026 Apr 13;16(21):19366–78. doi: 10.1039/d6ra01928e (PMC13075472; doi:10.1039/d6ra01928e)
Supplement: RA-016-D6RA01928E-s001 [file RA-016-D6RA01928E-s001.pdf]

Supplementary Information (SI) for RSC Advances.
This journal is © The Royal Society of Chemistry 2026

Supplementary

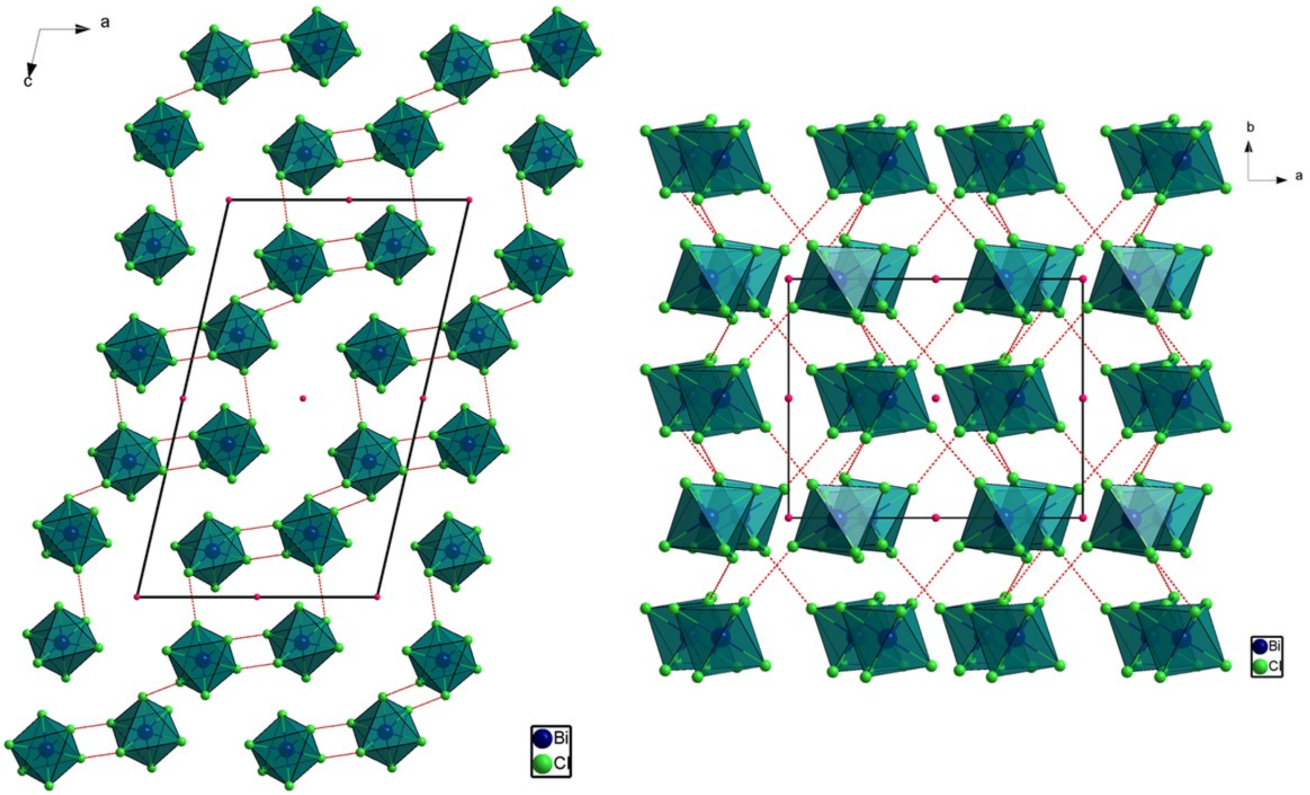

Fig. 1.S: halogen-halogen interactions arrangement

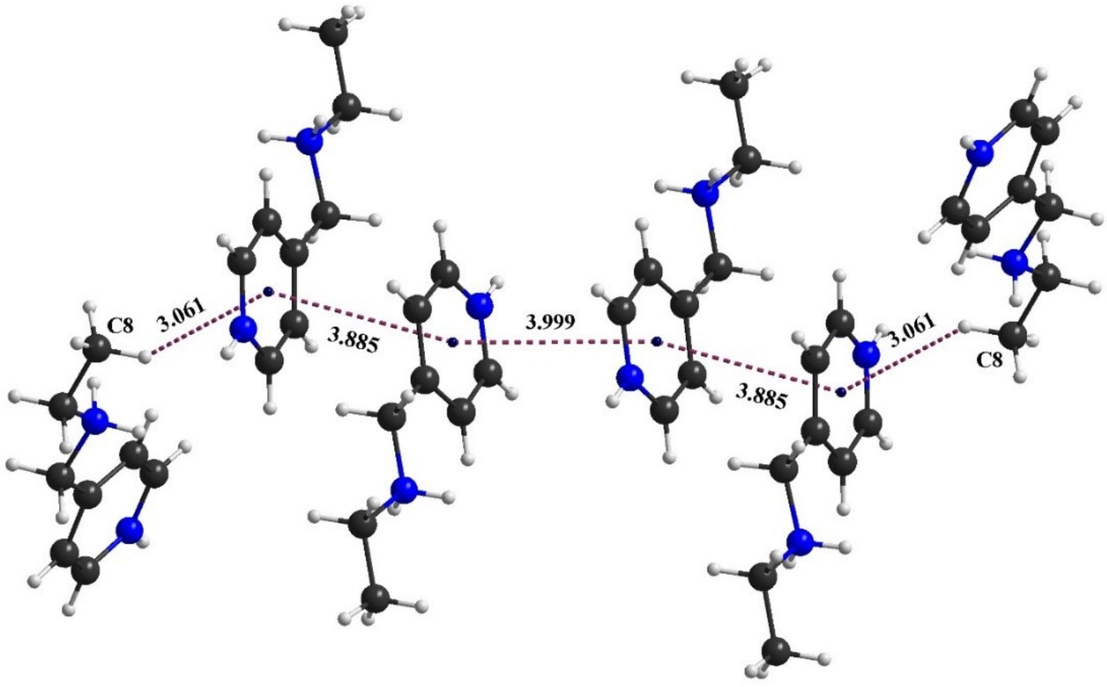

Fig. 2.S: $\pi \ldots \pi$ interactions

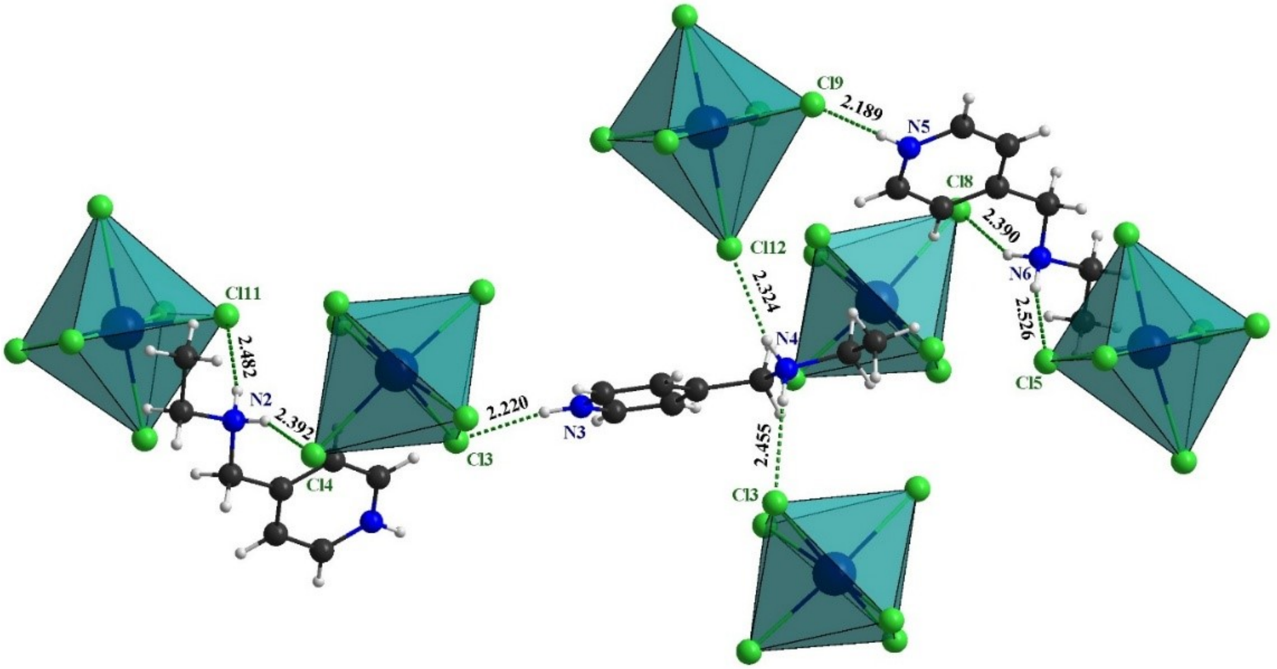

Fig. 3.S: Hydrogen bonding of the titled compound

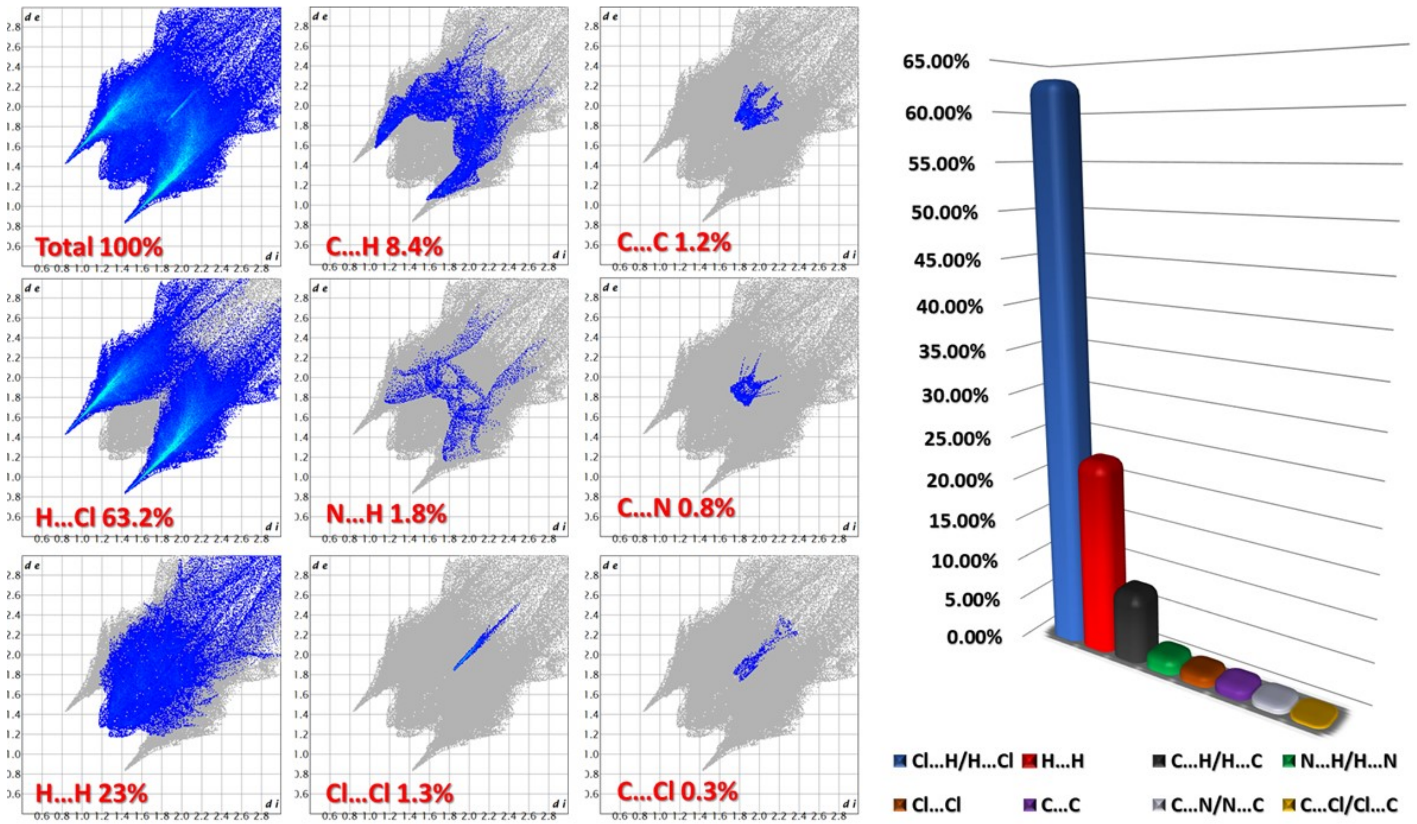

Fig. 4.S: 2D Visualization of Total Contact Contributions to the Hirshfeld Surface
